# Efficient DNA Repair Mitigates Replication Stress Resulting in Less Immunogenic Cytosolic DNA in Radioresistant Breast Cancer Stem Cells

**DOI:** 10.3389/fimmu.2022.765284

**Published:** 2022-02-25

**Authors:** Felix Meyer, Anna Maria Engel, Ann Kristin Krause, Tim Wagner, Lena Poole, Anna Dubrovska, Claudia Peitzsch, Kai Rothkamm, Cordula Petersen, Kerstin Borgmann

**Affiliations:** ^1^ Laboratory of Radiobiology & Experimental Radiooncology, Department of Radiotherapy and Radiation Oncology, Center of Oncology, University Medical Center Hamburg-Eppendorf, Hamburg, Germany; ^2^ OncoRay-National Center for Radiation Research in Oncology, Faculty of Medicine and University Hospital Carl Gustav Carus, Technische Universität Dresden, Helmholtz-Zentrum Dresden-Rossendorf, Dresden, Germany; ^3^ Helmholtz-Zentrum Dresden-Rossendorf, Institute of Radiooncology-OncoRay, Dresden, Germany; ^4^ German Cancer Consortium (DKTK), partner site Dresden and German Cancer Research Center (DKFZ), Heidelberg, Germany; ^5^ National Center for Tumor Diseases (NCT), Partner Site Dresden: German Cancer Research Center (DKFZ), Heidelberg; Faculty of Medicine and University Hospital Carl Gustav Carus, Technische Universität Dresden, and Helmholtz-Zentrum Dresden-Rossendorf (HZDR), Dresden, Germany; ^6^ Department of Radiotherapy and Radiation Oncology, Center of Oncology, University Medical Center Hamburg-Eppendorf, Hamburg, Germany

**Keywords:** immunogenic cytosolic dsDNA, radioresistance, replication stress, ATR inhibition, cellular immuneresponse, DNA repair, homologous recombination, breast cancer stem cells (BCSCs)

## Abstract

Cancer stem cells (CSCs) are a major cause of tumor therapy failure. This is mainly attributed to increased DNA repair capacity and immune escape. Recent studies have shown that functional DNA repair *via* homologous recombination (HR) prevents radiation-induced accumulation of DNA in the cytoplasm, thereby inhibiting the intracellular immune response. However, it is unclear whether CSCs can suppress radiation-induced cytoplasmic dsDNA formation. Here, we show that the increased radioresistance of ALDH1-positive breast cancer stem cells (BCSCs) in S phase is mediated by both enhanced DNA double-strand break repair and improved replication fork protection due to HR. Both HR-mediated processes lead to suppression of radiation-induced replication stress and consequently reduction of cytoplasmic dsDNA. The amount of cytoplasmic dsDNA correlated significantly with BCSC content (p=0.0002). This clearly indicates that HR-dependent avoidance of radiation-induced replication stress mediates radioresistance and contributes to its immune evasion. Consistent with this, enhancement of replication stress by inhibition of ataxia telangiectasia and RAD3 related (ATR) resulted in significant radiosensitization (SER37 increase 1.7-2.8 Gy, p<0.0001). Therefore, disruption of HR-mediated processes, particularly in replication, opens a CSC-specific radiosensitization option by enhancing their intracellular immune response.

## Introduction

Accumulation of DNA in the cytoplasm in the cell activates the innate immune response through cyclic GMP-AMP synthase (cGAS) and binding to the activator protein stimulator of interferon genes (STING). STING induces phosphorylation and translocation of the transcription factor interferon regulatory factor 3 (IRF3) and initiates the expression of type-1 interferon (type-1 IFN). This intracellular immune response primarily serves to defend against foreign DNA but cannot distinguish it from its own cytosolic DNA. The accumulation of self-DNA in the cytosol is triggered by DNA damage and leads to the production of type-1 IFN (1). The trigger for the increased occurrence of cytosolic DNA may be a defect in DNA repair mechanisms (2, 3). This has been observed when a defect in the DNA repair pathway homologous recombination (HR) is present (3–5). Increased accumulation of cytosolic DNA and activation of the cGAS/STING pathway have also been observed in RAD51-, BRCA1-, or BRCA2-deficient carcinoma cell lines. HR is the major DNA double-strand break repair pathway of the S phase. It serves to repair direct and replication-associated DNA double-strand breaks (DSBs) in an error-free manner. In addition, factors of HR, such as RAD51, BRCA1 and BRCA2, stabilize DNA at active replication forks and protect it from degradation by nucleases such as MRE11 (6, 7). This mediates repair and restart of replication forks, prevents formation of single-ended replication-associated DSBs, and thus avoids DNA replication stress. HR is activated by the kinases Ataxia telangiectasia and Rad3-related (ATR) and checkpoint kinase 1 (CHK1). ATR is recruited to replication protein A (RPA)-bound ssDNA, which occurs at DNA replication forks in the presence of DNA damage or dNTP deficiency and at resected DNA DSBs. ATR phosphorylates CHK1 and initiates the intra-S phase checkpoint. This leads to cell cycle arrest, prevents further firing of replication origins, and CHK1 is itself also involved in protecting stalled replication forks (8, 9). Through phosphorylation of BRCA2 and RAD51, CHK1 directly initiates HR-mediated DNA repair (10). Recent studies showed that disrupting the S-phase damage response by inhibiting ATR significantly increased the amount of cytosolic DNA after irradiation in breast cancer cells (11). Thus, the S-phase DNA damage response and DNA repair by HR to avoid DSB and replication stress are critical factors for the activation of the intracellular immune response.

Tumors are composed of a heterogeneous population of cancer cells with diverse replicative, tumorigenic, metastatic, and therapy-resistant capabilities. In particular, highly plastic subpopulations of stem-like cells within the tumor bulk, termed cancer stem cells (CSCs), tumor initiating cells (TICs) or tumor stem cells (TSC) have been described for breast cancer and are now considered to drive tumorigenesis, chemoresistance, and metastasis. This is mainly attributed to their upregulated DNA damage response and DNA repair capacity. Their radiosensitivity directly correlated with the number of CSCs in xenograft tumor models (12). In fact, repeated irradiation even led to an accumulation of CSC *in vitro* and *in vivo* in HNSCC, breast cancer, glioblastoma, and pancreatic cancers (13–18). It has long been assumed that CSC, just like tissue stem cells, are mostly in a quiescent state and DNA damage is mainly repaired by classical non-homologous end-joining (cNHEJ) (19). However, for CSC in glioblastoma and breast cancer, it has been shown that only about one third of CSC are quiescent and re-enter into the cell cycle after irradiation (16, 20). In fact, a higher proportion of S/G2 phase in CSC of triple-negative breast cancer (TNBC) compared to bulk cells was observed (21). Controversial experimental data are available about the contribution of cNHEJ to radiation resistance of CSC. So far, only an increased activation of DNA-dependent protein kinase (DNA-PKcs) after irradiation has been observed in glioblastoma CSC (22, 23). Other studies, however, showed a decreased activation of DNA-PKcs and ataxia telangiectasia mutated protein (ATM) after irradiation in CSC of NSCLC or a generally decreased cNHEJ activity in glioblastoma CSC (24, 25). Most studies observed a key role of the intra-S-phase kinase CHK1 in radiation resistance in glioblastoma CSC and breast cancer (14, 15, 26, 27). Increased expression of CHK1 was shown (14, 26, 27), as well as significantly stronger phosphorylation after irradiation (15, 26, 27). Phosphorylation of CHK1 resulted in cell cycle arrest and activated DNA repair by HR (28). Several studies demonstrated a dependence of CSC on HR and its key protein RAD51 (29). Glioblastoma CSCs showed high protein expression of RAD51 and dependence of CSC on HR repair after irradiation. Accordingly, the protein expression of RAD51 significantly decreased during differentiation (30). Correspondingly, inhibition of RAD51 resulted in significant radiation sensitization of glioma CSC (31). ALDH1-positive CSC of TNBC also showed increased RAD51 protein expression compared to ALDH1-negative cells, resulting in resistance to olaparib (32). After irradiation, isolated CSC from TNBC culture showed significantly more RAD51 foci than bulk culture (21). It is unclear what role ATR plays in this context, as CHK1 is one of the major downstream targets of ATR. ATR initiates cell cycle checkpoint, both during normal progression and in response to DNA damage. Therefore, most previous observations of BCSC resistance mechanisms suggest more effective DNA repair during replication mediated by CHK1 and its upstream kinase ATR.

In addition to a more effective DNA repair capacity, mechanisms of immune evasion were observed in CSC. A decreased expression of the antigen processing gene-associated transporter (TAP) and the co-stimulatory molecule CD80 was observed in ALDH1-positive BCSC, resulting in decreased susceptibility to T cells (33). Furthermore, an increased expression of PD-L1 was observed, which also suppresses T cell stimulation (34). Recently, it was also shown that DNA-damage in S-Phase leads to the activation of cGAS/STING pathway and further increases the expression of PD-L1, counteracting T-cell stimulation by the innate immune response. This was attributed to the activation of the ATR-CHK1 signaling pathway, leading to expression of the IRF1 gene *via* STAT3 and STAT1 phosphorylation, which resulted in increased PD-L1 gene expression (35). Thus, there appears to be a direct link between DNA damage response and immune evasion triggered by HR-mediated processes and activation of DNA damage response in S phase. The observations further imply that the innate immune response, particularly in BCSC, should be exploited by inhibiting its effective DNA repair mechanisms to successfully employ novel immunotherapies. This question is the subject of the presented study and was investigated using three TNBC breast cancer cell lines, a luminal reference cell line, their isogenic radioresistant clones, and isolated ALDH1-positive CSC.

## Materials and Methods

### Cell Culture and Treatments

All cell lines used in the study were either purchased from the American Type Culture Collection (ATCC, Manassas, VA, USA) or kindly provided by Prof. Dr. H. Wikman. The MCF7 is of the luminal subtype, the MDA-MB-231 is of the TNBC subtype. The MDA-231 BR (Brain) and -SA (Sarcoma) are derivatives of the MDA-MB-231 which were originally selected with respect to their metastatic behavior in xenograft (36, 37). In Xenografts they induce a primary tumor and only brain- (MDA-MB-231 BR) or only bone metastases (MDA-MB-231 SA). All cell lines were cultivated in DMEM medium with 10% FCS, 2% glutamine and 1% penicillin streptomycin in incubators at 37°C, 5% CO_2_ atmosphere and 100% humidity in cell culture flasks. ATR-inhibition was achieved by using the small molecule inhibitor VE-821 at 2 µM for 2h, for the inhibition of CHK1 the small molecule inhibitor MK-8776 was used at 2µM for 2h.

### Generation of Radioresistant Clones

Cells were irradiated with 4 Gy X-rays (200 kV, 1.2 Gy/min), surviving cells were pooled, cultivated for 10-14 days and irradiated again. This procedure was repeated 10 times to a total dose of 40 Gy. Radiosensitivity was checked 14 and 42 days after the last irradiation.

### Homologous Recombination Assay

HR capacity was measured by stable or transient transfection of the pDR-GFP (Addgene #26475, kindly provided by M. Jasin) plasmid, linearized by digestion with I-SceI enzyme prior to transient transfection. Briefly, 1 µg of linearized plasmid (pDR-GFP) was transfected into cells with FuGENE (Roche) at a ratio of 1:3 µg/µl according to the manufacturer’s instructions. In cells with stably integrated pDR-GFP 1µg I-SceI plasmid was transfected with FuGENE (Roche) at a ratio of 1:3 µg/µl. To measure transfection efficiency, cells were transfected with pEGFP-N1 (1 µg) in a parallel approach. After 24 hours, cells were harvested, and the percentage of GFP-positive cells determined by flow cytometry. HR capacity was calculated according to GFP-positive cells (pDR-GFP) and transfection efficiency (pEGFPN1) [**Supplementary Figure S2D** (38, 39)].

### DNA Fiber Assay

Exponentially growing cells were pulse labeled with 25 μM CldU (Sigma) followed by 250 μM IdU (Sigma) for 30 min each. Hydroxyurea (HU) was added for 4h between both labels. Labeled cells were harvested, DNA fiber spreads prepared and stained as described (40). Fibers were examined using an Axioplan 2 fluorescence microscope (Zeiss). CldU and IdU tracks were measured using ImageJ (40). At least 300 forks per sample were analyzed.

### Clonogenic Survival

250 cells per well were seeded in a 6-well plate 6h before irradiation and were cultured for 14 days. Cells were fixed and stained with 1% crystal violet in ethanol (Sigma-Aldrich, St. Louis, MO). Colonies with more than 50 cells were counted and normalized to untreated samples. Each survival curve represents the mean of at least three independent experiments.

### Immunofluorescence

Cells were seeded on culture slides. Cells were pulse labeled with 10 µM EdU for 20 minutes prior to treatment. After treatment the cells were fixed, permeabilized and blocked. Foci were detected using anti-53BP1 (Rabbit-anti 53BP1, 1:2000, Novus Biologicals), RPA (Mouse-anti RPA, 1:400, Santa Cruz), yH2AX (Rabbit-anti yH2AX, 1:250, Novus Biologicals), RAD51 (Rabbit, 1:500, Calbiochem), IFN-ß1 (Rabbit-anti IFN-ß1, 1:1000, Cell signaling) or IRF3 (Rabbit-anti IRF3, 1:400, Cell Signaling) followed by Alexa Fluor 488 goat anti rabbit IgG (Cell Signaling, 1:600), AlexaFluor 488 goat anti mouse IgG (Cell signaling, 1:500), AlexaFluor 594 goat anti rabbit IgG (Abcam, 1:600) or AlexaFluor 647 goat anti rabbit IgG (Cell Signaling, 1:600) and mounted (Vector Laboratories). EdU was stained with Alexa Fluor Azide 594 (Life Technologies, 1:500) and nuclei were stained with DAPI. Foci and fluorescence Intensity were quantified manually by capturing fluorescence images using a Zeiss Axioplan 2 fluorescence microscope equipped with a charge-coupled device camera and Axiovision software followed by quantification by Image J software. RPA/yH2AX-Foci were quantified automatically by the Aklides^®^-system (Medipan). Foci and fluorescence intensities of 100 cells per dose per slide and experiment were quantified.

### Flow Cytometric Analysis of CD44^high^/CD24^low^ Cells

Cells were harvested and washed in phosphate-buffered saline (PBS) with 0.5% fetal bovine serum. Combinations of fluorochrome-conjugated monoclonal antibodies against CD44 [APC, DB105, Miltenyi Biotec, 130-095-177 (1:100)] and unconjugated CD24 [CD24-biotin, eBioSN3 (SN3 A5-2H10), eBioscience, 13-0247-80 (1:50), followed by Alexa Fluor 405 (Cell signaling, 1:500)] were used. Primary antibodies or the respective isotype controls (BD Biosciences) were added to the cell suspension, as recommended by the manufacturer, and incubated at 4°C in the dark for 20 min. The labeled cells were analyzed *via* flow cytometry.

### Flow Cytometric Analysis of ALDH1-Activity

Cells were harvested, washed in PBS, incubated with ALDEFLUOR™ reagent (StemCell Technologies, Grenoble, France) and incubated at 37˚C for 45 minutes. Meanwhile, 5 μl of diethylaminobenzaldehyde (DEAB), a specific ALDH inhibitor, was added to 0.5 ml of ALDEFLUOR™-stained cells as a negative control. ALDH1-positive cells were then quantified by flow cytometry.

### PicoGreen^®^ Assay

Cells were irradiated with 8 Gy, harvested after 16 hours, washed in cold PBS and incubated with protease-inhibitors (Thermo Scientific Halt™ Protease Inhibitor Cocktail). Nuclear and cytoplasmatic fractions were separated with a nuclear and cytoplasmatic extraction reagent (Thermo Scientific NE-PER™). Cytoplasmatic dsDNA was stained using the Quant-iT™ PicoGreen^®^ dsDNA Reagent and Kits (Thermo Scientific). A standard curve was prepared and measured together with the samples in a Spark^®^ Microplate reader.

### Statistical Analysis

Statistical analysis, curve fitting and graphs were performed using Prism 6.02 (GraphPad Software). Data are given as mean (+SEM) of 3-5 replicate experiments. Unless stated otherwise, significance was tested by Student’s t-test.

## Results

### Cytosolic DNA Correlates With Breast Cancer Cell Proportion (BCSC)

**Figure 1:**
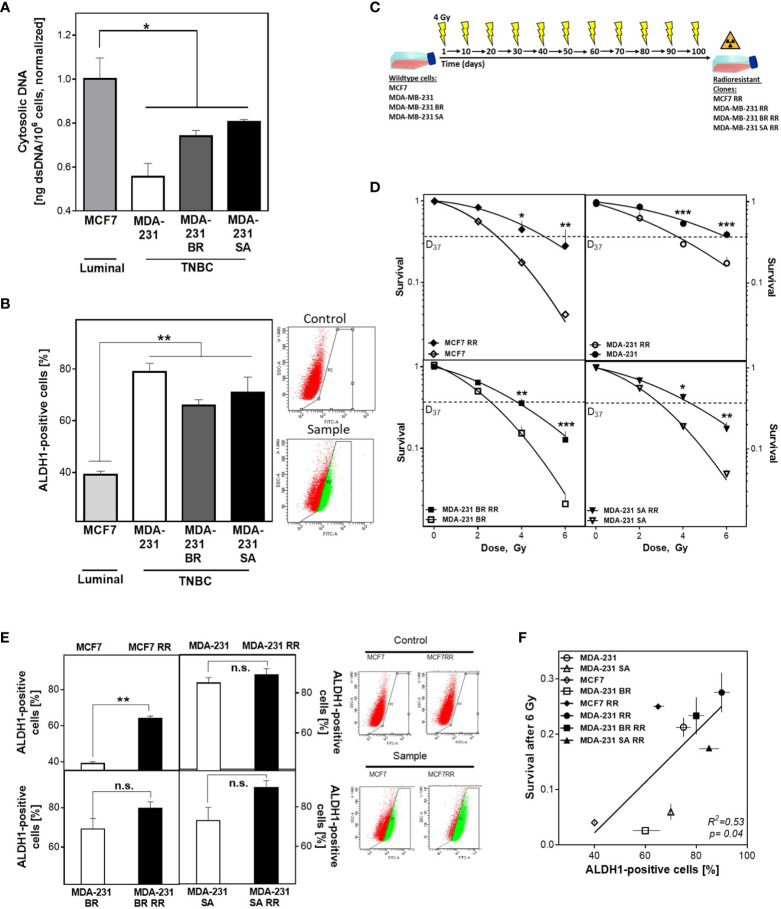
BCSC proportion correlates with cytosolic DNA and radioresistance. **(A)** Cytosolic dsDNA in TNBC and luminal cells. Cytoplasmatic fractions were isolated, dsDNA stained with PicoGreen^®^reagent and quantified in a Spark^®^ reader. **(B, E)** Detection of ALDH1 positive cells. Cells were treated with ALDEFLUOR™ reagent, harvested and the ALDH1 positive cells quantified by FACS. **(C)** Scheme to generate radioresistant sub cell lines. Cells were irradiated, pooled, and irradiated again after two weeks. The procedure was repeated ten times. **(D)** Cellular survival after irradiation. Cells were seeded 6 hours prior to treatment, irradiated with indicated doses, fixed after 14 days and the numbers of colonies was counted. **(F)** Correlation of the percentage of ALDH1-positive cells and cellular survival. Shown are means from three independent experiments ± SEM. Asterisks (*) represent significant differences (n.s., not significant; *p < 0.05; **p < 0.01; ***p < 0.001; Student’s t-test).

The appearance of cytosolic dsDNA is crucial for the initiation of the intracellular immune cascade (41). Cells with a DNA repair defect in HR experience increased activation of the cGAS/STING pathway and subsequent activation of the intracellular immune response due to elevated cytosolic dsDNA (4). It has not been investigated whether CSCs can suppress the induction of cytosolic dsDNA and thus an intracellular immune response, through efficient DNA repair mechanisms. To this end, the importance of the DNA damage response in relation to CSC content on radioresistance and the appearance of cytosolic dsDNA was investigated in three isogenic, triple-negative (TNBC) and one luminal cell lines and their corresponding radiation-resistant subclones. **Figure 1A** shows that TNBC cell lines have lower amounts of cytosolic dsDNA after than the luminal cell line, with only 0.55 ± 0.06, 0.74 ± 0.02 and 0.8 ± 0.01 in the MDA-MB-231 WT/BR/SA compared to the amount observed in MCF7 cells (p=0.04).

To investigate the relevance of the proportion of BCSC for this observation, ALDH1 activity was determined using the ALDEFLUOR™ assay (**Figure 1B**). TNBCs had almost twice as many ALDH1-positive cells compared to MCF7 cells (73 ± 6 versus 39 ± 1%, p<0.001), while the three TNBC cell lines examined had comparable proportions. The observed differences in CSC proportion were confirmed by further CSC markers such as plating efficency, migration ability and the proportion of CD44high/CD24low cells (**Supplementary Figures S1A–C**).

To further increase the proportion of BCSC, cell lines were repeatedly treated with ionizing radiation (**Figure 1C**), and the effects for cellular survival were analyzed (**Figure 1D**) (13, 14). Consistent with the assumption that the proportion of ALDH1-positive cells determines radiosensitivity, the initial cell lines already showed significant differences in radiosensitivity corresponding to their ALDH1-positive proportion. Accordingly, the radioresistant subclones (RR clones) of each cell line showed a marked increase in radioresistance compared to the baseline cell lines, with an increase in D37 between 1.2-1.8 (**Figure 1D** and **Figure S1D**). To confirm that radioresistance was due to the proportion of ALDH1-positive BCSCs, the ALDEFLUOR™ assay was performed (**Figure 1E**). As expected, all RR clones showed an increase in the proportion of ALDH1-positive cells. MCF7 cells showed the highest increase, approximately 25%, whereas TNBC cell lines showed only a slight increase in the already high proportion in the parental cell lines, ranging from 5% to 20%. Thus, a high proportion of ALDH1-positive BCSCs resulted in a lower incidence of cytosolic dsDNA and was consistent with the generally accepted concept that the more BCSCs present, the higher the radiation resistance (**Figure 1F**).

### Radiation Resistance Of BCSC Is Mainly Mediated In S Phase

**Figure 2:**
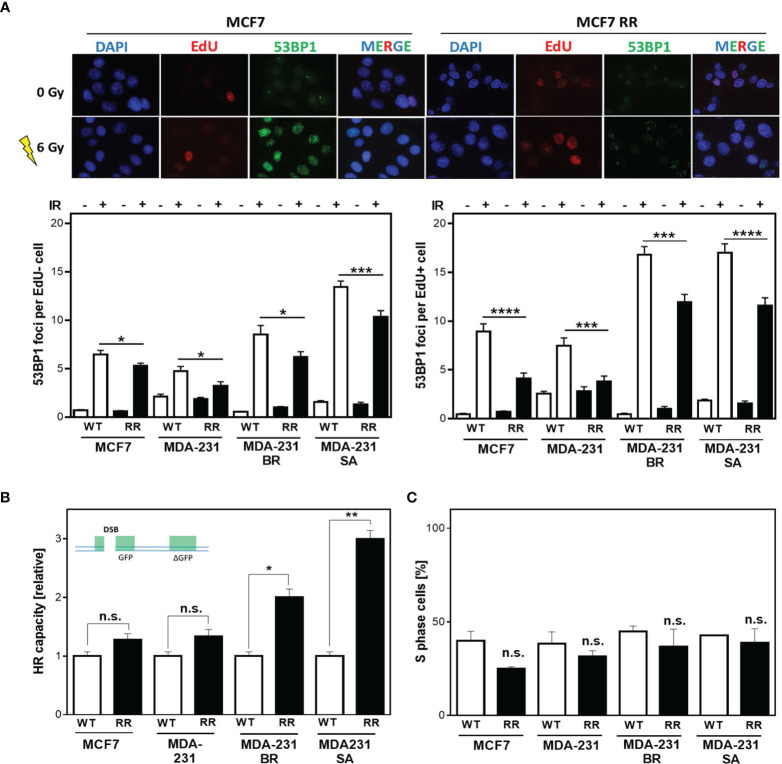
Radiation resistance of BCSC is mediated in S phase. **(A)** 53BP1 foci (green) in non-S phase (EdU-, bottom left) and S phase (EdU+, bottom right) cells after irradiation. Cells were irradiated with 6 Gy after pulse labeling with 10 µM EdU for 20 min. Immunostaining was per-formed 24h after treatment with a specific antibody against 53BP1 and a fluorescent second anti-body. Nuclei were stained with DAPI, replicating cells were discriminated by incorporated EdU stained with the “click-it”-reaction. Foci were quantified with Image J Software for EdU+ and EdU- cells (n = 100). **(B)** HR repair of DSB. Cells were transiently transfected with the linearized DR-GFP plasmid for 24h. The number of GFP-expressing cells was analyzed by FACS and HR capacity of the radioresistant clones was normalized to the absolute HR capacity of the parental cell lines. **(C)** Percentage of S Phase cells. Exponentially growing cells were pulse-labeled with 10 µM EdU for 20 minutes, fixed and EdU stained with the “click-it” reaction. Nuclei were counterstained with DAPI. The number of EdU+ and EdU- cells was counted (n=100). Shown are means of three independent experiments ± SEM. Asterisks (*) represents significant differences (n.s., not significant; *p < 0.05; **p < 0.01; ***p < 0.001, ****p < 0.0001, Student’s t-test).

Radiation sensitivity is significantly influenced by DNA repair in addition to other factors such as proliferation, cell cycle distribution. Therefore, it was tested whether the observed radioresistance of ALDH1-positive cells was due to enhanced DNA repair. **Figure 2A** shows examples (top) and quantification (bottom) of the number of 53BP1 foci remaining after 24 h in cells that were either outside of S phase (EdU-, **Figure 2A** bottom left) or actively replicating (EdU+, **Figure 2A** bottom right) at the time of irradiation with 6 Gy. All radioresistant clones showed significantly fewer 53BP1 foci after irradiation than the parental cell lines. This difference was even more pronounced when the cells were in S phase during irradiation. Here, MCF7/RR and MDA-MB-231/RR showed the strongest reduction in 53BP1 foci compared to their parental cell lines with 4.1 ± 0.57 vs. 8.9 ± 0.81, (p<0.0001) for MCF7/RR and 3.8 ± 0.56 vs. 7.3 ± 0.79 for MDA-MB-231/RR (**Figure 2A**, bottom right). MDA-MB-231BR/RR and -SA/RR also showed a significantly lower number of 53BP1 foci than the respective parent cell line with 11.9 ± 0.8 vs. 16.8 ± 0.9, (p=0.0001) and 11.6 ± 0.8 vs. 17.0 ± 0.9, (p<0.0001). Even in cells that were not in S-phase at the time of irradiation (**Figure 2A**, bottom left), enhanced DNA repair was also detected in the RR clones, but to a significantly lower level, with 5.3 ± 0.3 vs. 6.5 ± 0.4 for MCF7/RR (p=0.02), 3.2 ± 0.4 vs. 4.8 ± 0.5 for MDA-MB-231/RR (p<0.05), 6.2 ± 0.6 vs. 8.5 ± 0.9, for MDA-MB-231BR/RR (p=0.02) and 10.4 ± 0.7 to 13.5 ± 0.6, MDA-MB-231SA/RR (p=0.0007).

These results suggest that all, but especially the DNA repair pathways in S phase are upregulated in the RR clones. Since DSBs in S phase are mainly repaired by HR, investigations were focused on the analysis of HR-dependent processes. All cell lines examined showed HR competence, evident from the successful formation of RAD51 foci formation after treatment with mitomycin C (MMC) (**Supplementary Figure S2A**) as well as successful DNA repair of the HR specific reporter construct, after both transient and stable transfection (**Supplementary Figures S2B, C**) (39). Interestingly, in the RR clones significantly higher HR capacity compared to the respective parental cell line was observed, with 1.28 ± 0.08 vs. 1.05 ± 0.05 for MCF7/RR (n.s) and 1.34 ± 0.08 vs. 0.98 ± 0.05 for MDA-MB-231/RR (n.s). MDA-MB-231 BR-RR even showed a 2-fold and MDA-MB-231 SA-RR a 3-fold increase in HR capacity with 2.0 ± 0.1 vs. 1.01 ± 0.45, (p=0.012) and 2.95 ± 0.1 vs. 1.0 ± 0.07, (p=0.003), respectively (**Supplementary Figures S2B, C**) (39).

Since HR has its highest activity in S phase (28) it was important to ensure that the observed differences in HR capacity were not due solely to differences in cell cycle distribution in favor of increased S phase content in the RR clones. **Figure 2C** shows the percentage of S phase cells for the RR clones compared to the parental cell lines. It was apparent that the RR clones all had a lower S phase content than the parental cell lines, with 24.9 ± 1.1% vs. 40 ± 5.0% in MCF7, 31.6 ± 3.0% vs. 38.5 ± 6.2% in MDA-231, 36.8 ± 8.3% vs. 44.8 ± 3.0% in BR, and 38.9 ± 7.5% vs. 42.9 ± 0.5% in SA, which was not significant in any of the cell lines.

Taken together, these data indicate that the observed radioresistance is largely mediated by DNA repair processes involving HR in S phase through increased HR-dependent DNA repair in RR clones. It is unclear whether this is solely attributable to more efficient double-strand break repair (DSB repair) or whether the replication-associated functions of HR are of much greater importance.

### Avoidance Of Replication Stress By Functional HR Mediates Radioresistance Of BCSC

**Figure 3:**
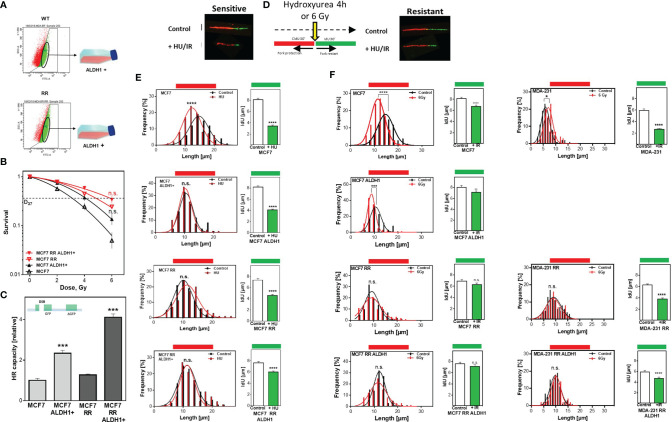
Avoidance of replication stress by functional HR mediates radiation resistance of BCSC. **(A)** Isolation of ALDH1 positive cells. Cells were treated with ALDEFLUOR™ reagent, harvested and ALDH1-positive cells isolated by FACS sorting. **(B)** Cellular survival after irradiation. Cells were seeded 6 hours prior to treatment, irradiated with the indicated doses, fixed after 14 days and the number of colonies was counted. **(C)** HR repair of DSB. Cells were transiently transfected with the linearized pDR-GFP construct for 24h. The number of GFP-expressing cells was analyzed by FACS and HR capacity of the ALDH1 positive cells was normalized to their respective parental cell lines. **(D–F)** Replication tract lengths after HU or irradiation. MCF7 cells were sequentially labelled with CldU and IdU for 30min and either treated with HU or MCF7 and MDA-MB-231 cells irradiated with 6 Gy between both labels. DNA was spread on slides, fixed, and incorporated nucleotides were detected by immunofluorescence. The lengths of the DNA fibers were measured with the Image J software. Shown are means of three independent experiments ± SEM. Asterisks (*) represent significant differences (n.s., not significant; *p < 0.05; **p < 0.01; ***p < 0.001, ****p < 0.0001, Student’s t-test).

To ensure that the increased radioresistance and HR capacity was attributable to the proportion of ALDH1-positive BCSC, they were isolated by FACS sorting from the parental MCF7 as well as the radioresistant MCF7 clone (**Figure 3A**) and their radiosensitivity was determined (**Figure 3B**). There was a comparable increase in radioresistance for both ALDH1-positive subpopulations to a D37 of 4.1 ± 0.1 compared to 3.0 ± 0.2Gy for the parental MCF7 cell line and for the already radioresistant subclone with a D37 to 5.8 ± 0.1 Gy compared to 4.8 ± 0.2 Gy. The same scenario was observed for HR capacity with an increase in HR capacity in both ALDH1-positive subpopulations (**Figure 3C**). A 2-fold increase in HR capacity was seen for the ALDH1-positive cells of the parental MCF7 with 2.3 ± 0.09 vs. 1.0 ± 0.06, p=0.0002 and a 4-fold increase in HR capacity compared to the RR clone with 4.1 ± 0.1 vs. 1.0 ± 0.05, p=0.0002.

In addition to the extensively described importance of HR for DSB repair, several studies showed that HR proteins play an essential role in stabilizing active replication forks and that their loss led to nucleolytic degradation (6, 7). To verify whether the increased HR capacity also translates into a stronger defense against nucleolytic degradation of active replication (6), both replication fork stability and restart after treatment with HU, which depletes the nucleotide pool without damage induction, were examined by the DNA fiber assay (**Figure 3D**). Parental MCF7 cells show a significant degradation of already synthesized DNA, which was manifested by significantly shorter chromatin fibers compared to the untreated control, with 11.9 ± .0.2µm vs. 14.7 ± 0.17 µm (p<0.0001) (**Figure 3E** left). In contrast, neither the radioresistant clone nor the two ALDH1-positive subpopulations showed pronounced degradation of the already synthesized DNA with 10.3 ± 0.12 vs. 10.5 ± 0.19 µm, 11.3 ± 0.32 vs. 10.8 ± 0.13 µm and 11.3 ± 0.36 vs. 11.1 ± 0.18µm, respectively. Moreover, these results surprisingly showed that the three subpopulations replicated significantly slower than the parent MCF7 cell line, with 0.84 ± 0.02 kb/min in MCF/ALDH1-positive cells, 0.81 ± 0.03 kb/min in the RR clone of MCF7 cells, 0.9 ± 0.03 kb/min in the ALDH1-positives of the RR clones compared to 1.06 ± 0.03 kb/min in wild type MCF7 cells, indicating that CSC-enriched populations exhibited significantly more endogenous replication stress than the baseline cell line.

Analysis of replication fork restart after HU removal (IdU labeling), another characteristic of functional HR (42), also showed significant differences (**Figure 3E** right). The longest time for replication restart was required by the parental cell line, evident by the shortest replication tracts with 3.4 ± 0.2 vs. 8.1 ± 0.3, (p<0.0001). Slightly faster, the ALDH1-positive cells of the parental MCF cells reached replication restart with a length of 4.1 ± 0.2 to 8.2 ± 0.2 (p<0.0001). Again, surprisingly, both the RR clone and the ALDH1-positive subpopulation derived from it were significantly faster capable to resume replication, with 4.5 ± 0.2 vs. 6.9 ± 0.3 (p<0.0001) and 6.2 ± 0.2 vs. 7.6 ± 0.2, (p<0.0001). After irradiation, a similar pattern is seen in both CldU shortening and replication restart (**Figure 3F**, left). MCF7 cells show significant shortening of CldU labeling, which is not observed in the RR clone of MCF7 cells or in ALDH1-positive cells of either line. Similarly, replication restart is significantly faster in the populations with increased CSC content, such as the ALDH1-positive MCF7 cells, the MCF7-RR clone, and the ALDH1-positive cells of the RR clone, as revealed by significantly longer IdU strands. Interestingly, the respective cell lines showed differences in replication restart depending on their radiosensitivity (**Figure 3F**, left), the more radioresistant, the longer the IdU strand. In MDA-MB-231 cells, which already have a CSC content of about 80% in the initial population (**Figure 1B**), the RR clone and ALDH1-positive cells of the RR clone show no pronounced irradiation effect on the length of the already synthesized DNA (**Figure 3F** right CldU labeling). However, there was a clear dependence between restart of DNA replication and radiation resistance: the longer the IdU tract, the higher the radiation resistance (**Figure 3F**, far right).

**Figure 4:**
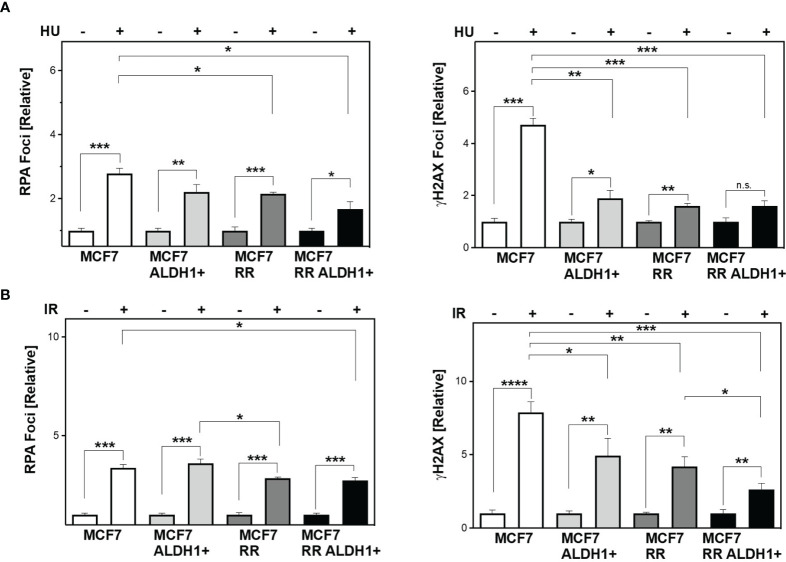
Lower DNA replication stress leads to less DSB in BCSC after treatment. Cells were incubated with HU **(A)** for 2h or irradiated with 6 Gy **(B)**. Immunostaining was performed 3h after treatment with a specific antibody against RPA and уH2AX and fluorescent secondary antibodies. Nuclei were stained with DAPI, quantification of the foci was performed by automatic foci detection in the Aklides^®^-system (Medipan). For each analysis the foci of at least 100 cells were quantified. Shown are means of three independent experiments ± SEM. Asterisks (*) represent significant differences (*p < 0.05; **p < 0.01; ***p < 0.001, ****p < 0.0001, Student’s t-test).

Next, the question was whether differences in the ability to protect active replication forks directly impacts the number of DSB. To this end, RPA and yH2AX were analyzed in parallel after treatment with HU and irradiation (**Figures 4A, B**). It was observed that stalled replication forks resulted in single-stranded DNA in all cell lines examined (RPA foci), but significantly less frequently in the ALDH1-positive with 2.2 ± 0.2 and the radioresistant clone with 2.14 ± 0.1 than in the parental cell line with 2.8 ± 0.2, with the ALDH1-positive population of the radioresistant clone having the lowest number of RPA foci with 1.7 ± 0.2.

In parallel, the number of DSB (yH2AX) also showed significantly lower values with increase in CSC content in the cell lines studied, with the difference from MCF to ALDH1-positive MCF7 cells being most pronounced with 4.7 ± 0.3 for WT MCF7 to 1.89 ± 0.3 and only slightly reduced in the RR clone and its ALDH1-positive cells at 1.61 ± 0.07 and 1.6 ± 0.19, respectively (**Figure 4A**, right), supporting the lower replication stress observed after HU in these cell lines (**Figure 3E**).

After irradiation, however, a different pattern emerges. While all cell lines examined showed a comparable number of RPA foci, those with an increase in CSC content showed a decrease in DSB 3 h after irradiation (**Figure 4B**).

### The Amount Of Cytosolic DNA Depends On The ALDH1-Positive BCSC Fraction

**Figure 5:**
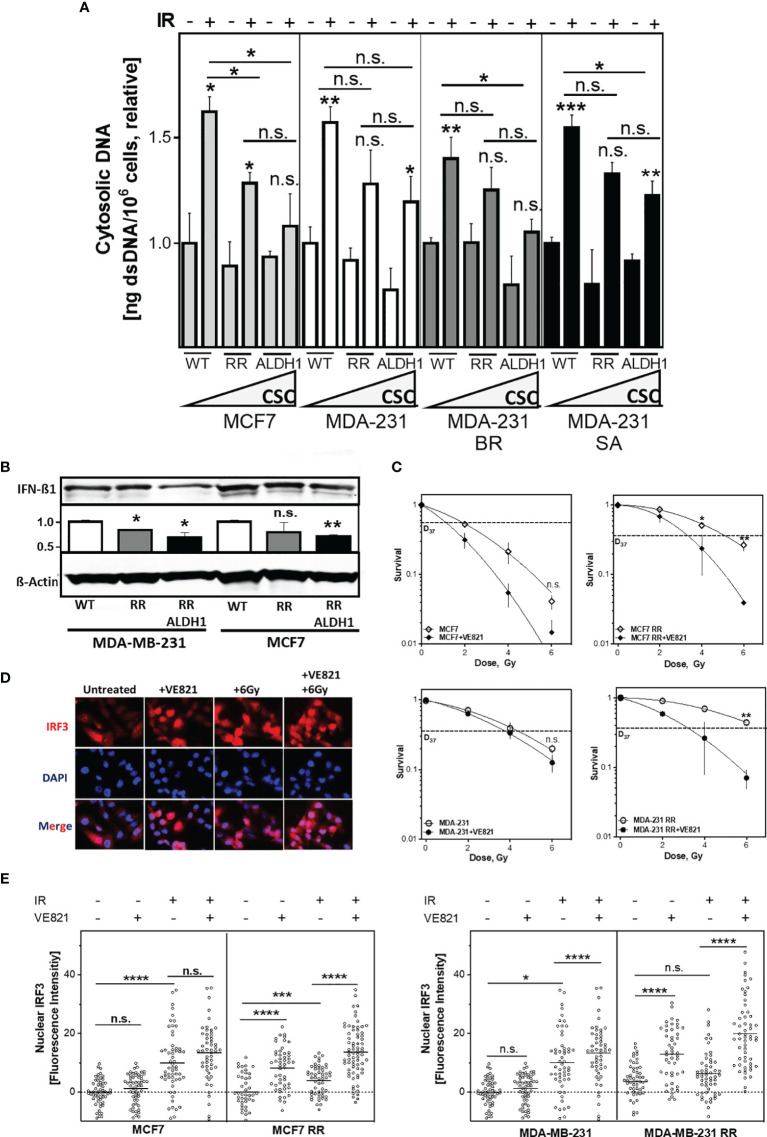
The amount of cytosolic DNA and intracellular immune response depends on the ALDH1-positive BCSC proportion. **(A)** Relative increase of cytosolic dsDNA after irradiation. Cells were irradiated with 8 Gy, cytoplasmatic fraction isolated and dsDNA stained with PicoGreen^®^reagent, quantified in a Spark^®^ reader and normalized to the untreated control or **(B)** Expression of IFN-ß1 after irradiation. Cells were irradiated with 8 Gy and proteins were extracted 24h later. Proteins were separated and transferred by Western blotting. IFN-ß was detected specific primary antibodies, followed by fluorescence-coupled secondary antibodies. ß-Actin served as a loading control. The Expression of IFN-ß was normalized to the wild type cell lines. **(C)** Cellular survival after irradiation. Cells were seeded 6 hours prior to treatment, treated with +/- VE821 2h prior to irradiation, irradiated with the indicated doses, fixed after 14 days and the number of colonies was counted. **(D, E)** Nuclear IRF3 accumulation. Cells were incubated with 1µM VE821 for 2h, irradiated with 6Gy and fixed 16h later. IRF3 was stained with a specific primary antibody, followed by a fluorescent secondary antibody. Nuclei were stained with DAPI. Fluorescence intensity (FI) of IRF3 was quantified with Image J Nuclear IRF3 was calculated by subtraction of the cytoplasmatic FI from the nuclear FI. Shown are means of three independent experiments ± SEM. Asterisks (*) represent significant differences (n.s., not significant; *p < 0.05; **p < 0.01; ***p < 0.001, ****p < 0.0001, Student’s t-test).

Next, it was of interest to determine whether the enhanced DNA repair capacity *via* HR of RR clones and their respective ALDH1-positive BCSC fractions affect the amount of cytosolic dsDNA after irradiation (**Figure 5A**). There was a significantly decreased accumulation in cytosolic dsDNA, both in the RR clone and their ALDH1-positive cells after irradiation compared with the parental cell line in all cell lines examined. Among them, MCF7 cell line showed the most obvious and MDA-MB-231 the smallest decrease of cytosolic DNA in ALDH1-positive cells compared to the baseline cell lines and their RR clones, from 1.62 ± 0.1 to 1.28 ± 0.1 and 1.08 ± 0.2, respectively, p<0.05 in MCF7 and 1.57 ± 0.1 in MDA-MB-231 to 1.28 ± 0.2 and 1.2 ± 0.1 in MDA-MB-231, respectively (n.s.). The other TNBC cell lines behaved in the same manner. Thus, irradiation led to an increase in cytosolic dsDNA in all cell lines, which became lower with increasing ALDH1-positive BCSC content. Indeed, the proportion of ALDH1-positive cells correlated significantly with the amount of cytosolic DNA after irradiation with R^2^ of 0.8, p<0.05 (**Supplementary Figures S3A, B**). Supporting this, IFN-ß1 showed a correspondingly lower expression in the ALDH1-positive cells compared to the RR clones and WT MCF7 and MDA-MB-231 cells after irradiation (**Figure 5B**).

DSB repair by HR and control of DNA replication stress are both dependent on the activation by the ATR-CHK1 pathway (10). To test whether radioresistance of the RR clones depended on the functionality of the S phase checkpoint, ATR was inhibited (**Figure 5C**). Notably, the RR clones were severely sensitized by ATR inhibition, whereas the parental cell lines showed only a moderate radiosensitization, with a reduction of the D37 about 1.7 Gy in the RR clone compared to only 1.0 Gy in the parental cell line, p=0.02 and p=0.004, respectively. p=0.002 (**Supplementary Figure S3C**). Additionally, the inhibition of the ATR downstream kinase CHK1 with the CHK1-inhibitor MK-8776 also led to a specific radiosensitization of the RR clones of the MCF7 and the MDA-MB-231, confirming the importance of the ATR-CHK1 signaling pathway to their radioresistance (**Supplementary Figure S3C**). Thus, radioresistant, ALDH1-positive BCSC are particularly dependent on the S phase damage checkpoint, HR-mediated DSB repair and replication fork protection.

Of particular interest was whether inhibition of ATR affects activation of the intracellular immune response after irradiation. Activation of the intracellular immune response by the appearance of cytosolic dsDNA occurs through pSTING phosphorylated and thereby activated IRF3, which is translocated by this process to the nucleus where it induces typeI IFN expression (43) (**Figures 5D, E**). As expected, irradiation alone in RR clones of both cell lines leads to a low translocation of IRF3 into the nucleus (p=0.006 and n.s.), whereas a significant increase was observed in the parental cell lines (p<0.0001 and p=0.02). Also here, the extent of nuclear IRF3 after irradiation correlated with the percentage of cytosolic DNA after irradiation (**Supplementary Figure S4A**). In contrast, inhibition of ATR alone led to a significantly higher translocation of IRF3 to the nucleus in RR clones compared to parental cells (both with p<0.0001), whereas only weak translocation of IRF3 was observed in the parental cell lines. The combined treatment of ATR inhibition with irradiation resulted in an additive increase in IRF3 translocation, with a significantly stronger expression in the RR clones (both p<0.0001). Thus, inhibition of ATR enhances the activation of intracellular immune response after irradiation in BCSC by suppressing their functional S-phase DNA damage response.

## Discussion

Here, we show that the increased radioresistance of ALDH1-positive BCSC in S phase is mediated by both enhanced DSB repair and improved replication fork protection due to HR. Both HR-mediated processes lead to suppression of radiation-induced replication stress and consequently reduction of cytoplasmic dsDNA. The amount of cytoplasmic dsDNA correlated significantly with BCSC content. This clearly indicates that HR-dependent avoidance of radiation-induced replication stress mediates radioresistance and contributes to its immune evasion. Consistent with this, enhancement of replication stress by inhibition of ATR resulted in significant radiosensitization. Therefore, disruption of HR-mediated processes, particularly in replication, opens a CSC-specific radiosensitization option by enhancing their intracellular immune response.

An abundance of CD44^high^/CD24^low^ and ALDH1 positive cells in the TNBC cell lines compared to the luminal MCF7 cell line was observed (**Figures 1B, E** and **Supplementary Figure S1C**). Ma and colleagues already showed an enrichment of CD44^high^/CD24^low^ cells in TNBC (44). This putative high proportion of BCSC is confirmed by work of others, who reported ~45% of CD44^high^/CD24^low^ cells in untreated TNBC and only ~5% in luminal A tumor biopsies (45, 46). Compared to Glioma with only ~2-4% of CD133+ CSC found in human specimens, the proportion in TNBC is enormous and clearly shows the relevance of BCSC in TNBC (15). For the identification of BCSC both the CD44^high^/CD24^low^ phenotype and ALDH1 activity are important, but CD44^high^/CD24^low^ is limited to a mesenchymal phenotype, whereas ALDH1 is a more general BCSC marker due to its independence from the current cell state (47, 48). This explains the higher proportion of ALDH1-positive cells in comparison to CD44^high^/CD24^low^ cells. A weakness of both markers is that they are also expressed by progenitor cells. To overcome this problem, others considered only the 1% of cells with the highest ALDH1-activity as CSC, the cells with the lowest 1% of ALDH1-activity as progenitor cells (49). Since both populations with ALDH1-activity remained tumorigenic, it was assumed - based on surface markers or ALDH1 activity – that there is no clear distinction between stem and progenitor cells.

Baumann and colleagues postulated that radiation resistance is due to the proportion of CSC (12). This is also confirmed by our data showing a clear correlation between radiation resistance and ALDH1-positive BCSC (**Figure 1F**).

It has been previously reported that repeated irradiation with fractions ranging from 4-6 Gy to a total dose of 12-56 Gy worked as a strategy to increase the endogenous CSC proportion in breast and HNSCC cell lines (13, 14). In line with this we achieved an increase in the ALDH1-positive CSC proportion and a significantly increased survival after irradiation in all investigated cell lines, independent of the molecular subtype (**Figures 1E, F**). This acquired radioresistance can be attributed to i) selection of pre-existing, radioresistant clones, ii) radiation-induced de-differentiation to a stem cell phenotype (17) and iii) alterations of DNA repair processes (14). We found that radioresistance was indeed determined by the ALDH1-positive cell fraction (**Figures 3A–C**). We also found that the ALDH1-positive cells from the MCF7 cell line were more radiosensitive than the radioresistant clone, suggesting that not only clonal selection, but also alterations of DNA repair processes due to repeated irradiation played a role. This is in line with observations in radioresistant BCSC showing a ZEB1 dependent stabilization of CHK1, mediating radioresistance (14).

It is generally believed that CSC, similar to tissue stem cells, are mostly quiescent (19), but studies in glioma- and breast cancer cell lines showed, that only one-third of the CSC were dormant, but entered the cell cycle after irradiation (16, 20). We found that in non-S phase cells the radioresistant clones had significantly lower amounts of residual DSBs than the parental cells after irradiation, suggesting increased DNA repair by NHEJ in BCSC (**Figure 2A**, EdU-negative). Even more striking was that these differences were even more significant when the cells were irradiated during S phase (**Figure 2A**, EdU-positive). In S phase HR is the most important DNA repair pathway for the repair of frank DNA-DSB and the avoidance of DNA replication stress by replication fork protection (39). Here, we demonstrate that HR capacity is greatly increased in in the RR clones due to the ALDH1-positive cell fractions (**Figures 2B**, **3C**). These effects were not due to cell cycle changes in favor of the S phase, the RR clones provided a slightly lower S phase proportion than the parental cell lines, so the actual HR capacity of the RR clones could be higher than depicted in the figure (**Figures 2B, C**) (28). However, we demonstrate here for the first time a functional stabilization of DNA replication forks in ALDH1-positive BCSC, which further supports the increased DSB repair by HR (**Figures 3C, D**). Other than the parental MCF7 cell line the ALDH1-positive BCSC showed no replication fork degradation after HU treatment and improved replication fork restart (7). Since a functional DNA damage response (DDR) is necessary for replication fork protection (50), these results compensate the potential lack of DDR activation for measuring the HR capacity with the plasmid reconstruction assay (51). This functional HR led to a lower occurrence of DNA-replication stress markers after HU and irradiation (**Figures 4A, B**) (52). Thus, our study demonstrates the importance of ATR and CHK1, avoiding degradation of nascent DNA strands. These findings further extend observations in glioma and breast CSC (14, 25, 29, 31). The S-Phase kinase ATR and its downstream kinase CHK1 activate HR (10, 53). These kinases also regulate DNA replication processes and support replication fork protection (50, 53–55). Others observed an increased expression and activation of CHK1 after irradiation in breast- and glioma CSC (14, 15, 27). Here we show that the inhibition of ATR and CHK1 lead to a significant radiosensitization of the RR clones (**Figure 5B** and **Supplementary Figures S3C, D**), suggesting a critical role of the ATR-CHK1 signaling cascade in preventing radiation-induced replication stress and protection of replication forks in BCSC. To our knowledge this is the first study that shows a targeted radiosensitization by ATR inhibition in BCSC. Yet, similar effects were only observed in CD133+ colon carcinoma stem cells, where ATR inhibition abrogated the tumorigenicity of CD133+ CSC (56). Thus, the activation of the ATR signaling cascade mediates radioresistance in BCSC by activating HR.

It has been previously shown that efficient DSB repair and avoidance of DNA replication stress by functional HR prevents the formation of radiation-induced cytosolic dsDNA (4). Consistent with this, we show that the proportion of ALDH1-positive BCSCs significantly affects the amount of cytosolic dsDNA after irradiation (**Figure 5A** and **Supplementary Figure S3B**). The resulting lower amount of cytosolic DNA led to decreased activation of the intracellular immune response, as evidenced by decreased nuclear IRF3 levels in the radioresistant BCSC (**Figure 5C** and **Supplementary Figure S4**). This suggests that upregulated HR processes protect BCSC not only from DNA damage, but also from the activation of the intracellular immune response. This would indirectly contribute to the CSC-specific mechanisms of immune escape and complement their enhanced expression of PD-L1 (33, 34). Consequently, disruption of HR by inhibition of ATR not only resulted in a specific radiosensitization of BCSC, but also in a significantly increased translocation of IRF3 to the nucleus, thus abrogating their low activation of the intracellular immune response after irradiation alone. This is in line with other *in vitro* and *in vivo* studies showing a significantly increased the activation of the immune response, expression of inflammatory genes and the infiltration of CD8+ T-cells after combination of irradiation with ATR inhibition in comparison to irradiation alone (11, 57, 58). Thus, the inhibition of the ATR signaling cascade specifically sensitizes BCSC to irradiation and increases the activation of the intracellular immune response, potentially overcoming CSC-mediated tumor protection.

## Data Availability Statement

The original contributions presented in the study are included in the article/**Supplementary Material**. Further inquiries can be directed to the corresponding author.

## Author Contributions

Conceptualization, KB and FM. Methodology, FM and AE. Software, FM. Validation, KB and FM. Formal analysis, FM and AE. Investigation, FM, AE, AK, TW. and ClP. Writing—original draft preparation, FM and KB. Writing—review and editing, KB, KR, CoP, and AD. Visualization, FM. Supervision, KB. Project administration, KB. Funding acquisition, KB. All authors have read and agreed to the published version of the manuscript.

## Funding

This research was funded by BMBF grant no. 02NUK032 and 02NUK035B, DFG grant no. BO1868/5, Wilhelm-Sander Foundation project 2017.106.1

## Conflict of Interest

The authors declare that the research was conducted in the absence of any commercial or financial relationships that could be construed as a potential conflict of interest.

## Publisher’s Note

All claims expressed in this article are solely those of the authors and do not necessarily represent those of their affiliated organizations, or those of the publisher, the editors and the reviewers. Any product that may be evaluated in this article, or claim that may be made by its manufacturer, is not guaranteed or endorsed by the publisher.
