# Supplementary material for: Efficient DNA Repair Mitigates Replication Stress Resulting in Less Immunogenic Cytosolic DNA in Radioresistant Breast Cancer Stem Cells
Source: Front Immunol. 2022 Feb 25;13:765284. doi: 10.3389/fimmu.2022.765284 (PMC8913591; doi:10.3389/fimmu.2022.765284)
Supplement: Supplementary file 1 [file Presentation_1.pptx]

## Slide 1
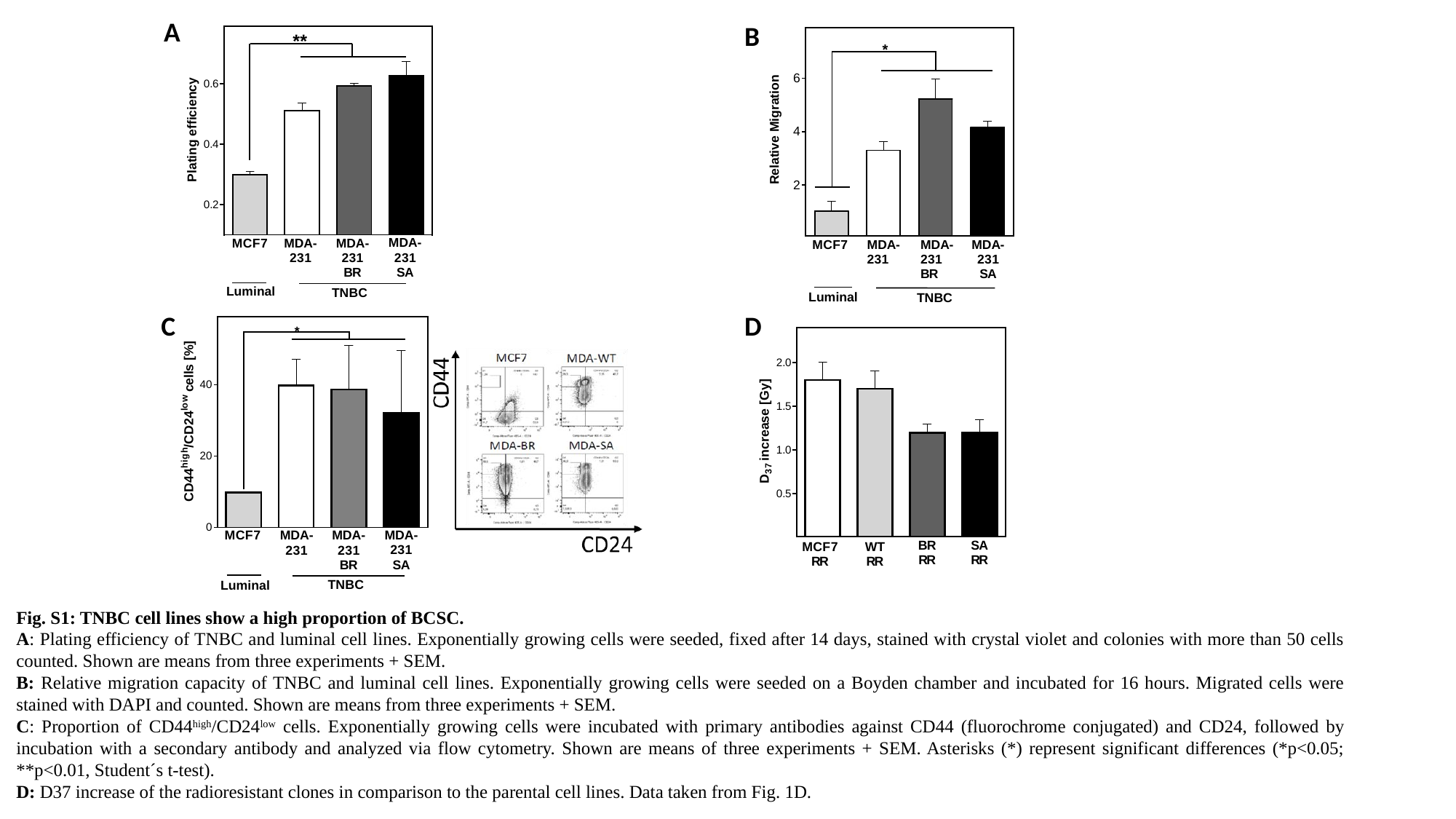

A
B
Luminal
TNBC
C
D
Fig. S1: TNBC cell lines show a high proportion of BCSC.
A: Plating efficiency of TNBC and luminal cell lines. Exponentially growing cells were seeded, fixed after 14 days, stained with crystal violet and colonies with more than 50 cells counted. Shown are means from three experiments + SEM.
B: Relative migration capacity of TNBC and luminal cell lines. Exponentially growing cells were seeded on a Boyden chamber and incubated for 16 hours. Migrated cells were stained with DAPI and counted. Shown are means from three experiments + SEM.
C: Proportion of CD44high/CD24low cells. Exponentially growing cells were incubated with primary antibodies against CD44 (fluorochrome conjugated) and CD24, followed by incubation with a secondary antibody and analyzed via flow cytometry. Shown are means of three experiments + SEM. Asterisks (*) represent significant differences (*p<0.05; **p<0.01, Student´s t-test).
D: D37 increase of the radioresistant clones in comparison to the parental cell lines. Data taken from Fig. 1D.

## Slide 2
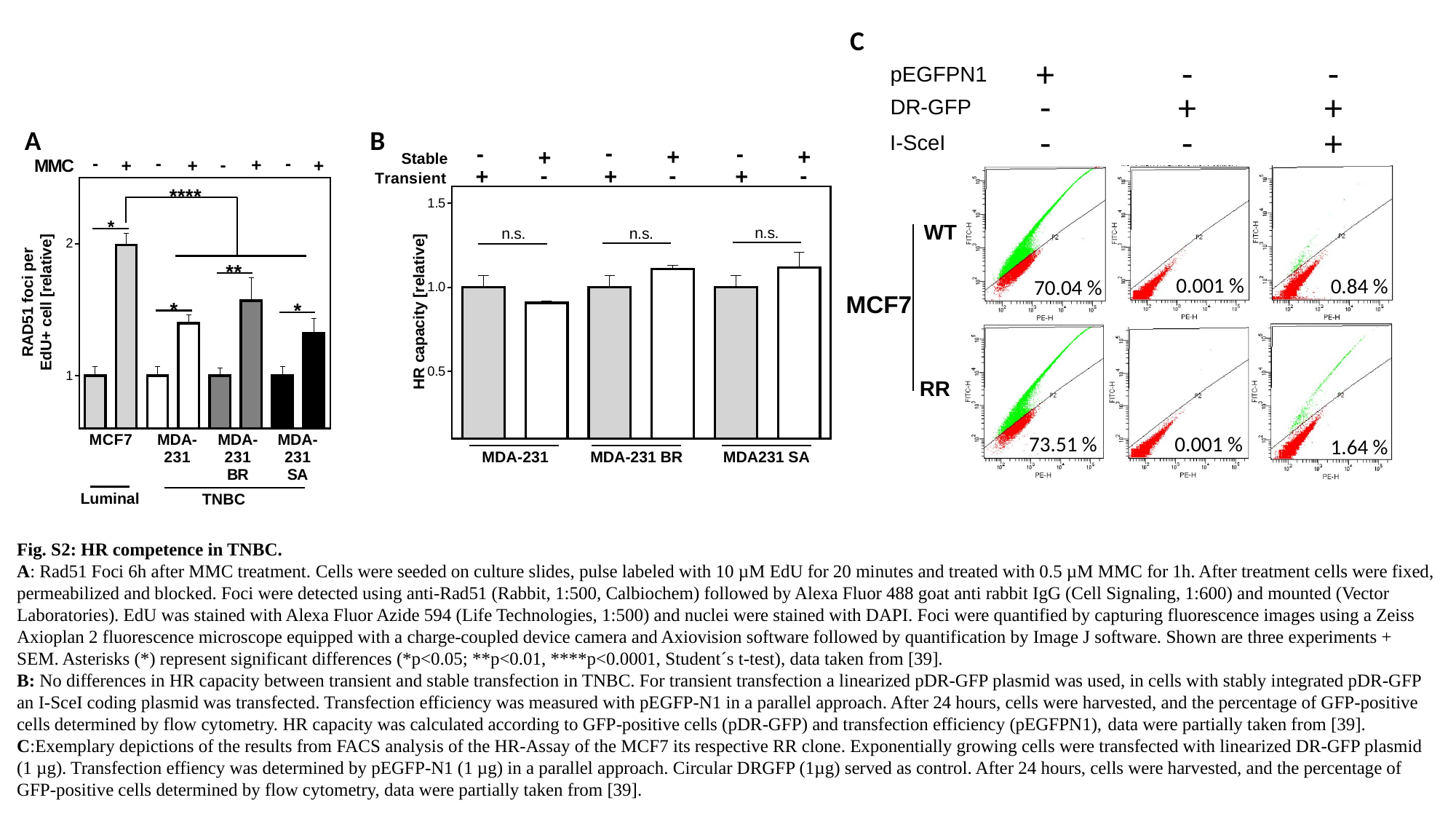

C
+
-
-
pEGFPN1
-
+
+
DR-GFP
-
-
+
A
B
I-SceI
WT
0.001 %
0.84 %
70.04 %
MCF7
RR
73.51 %
0.001 %
1.64 %
Fig. S2: HR competence in TNBC.
A: Rad51 Foci 6h after MMC treatment. Cells were seeded on culture slides, pulse labeled with 10 µM EdU for 20 minutes and treated with 0.5 µM MMC for 1h. After treatment cells were fixed, permeabilized and blocked. Foci were detected using anti-Rad51 (Rabbit, 1:500, Calbiochem) followed by Alexa Fluor 488 goat anti rabbit IgG (Cell Signaling, 1:600) and mounted (Vector Laboratories). EdU was stained with Alexa Fluor Azide 594 (Life Technologies, 1:500) and nuclei were stained with DAPI. Foci were quantified by capturing fluorescence images using a Zeiss Axioplan 2 fluorescence microscope equipped with a charge-coupled device camera and Axiovision software followed by quantification by Image J software. Shown are three experiments + SEM. Asterisks (*) represent significant differences (*p<0.05; **p<0.01, ****p<0.0001, Student´s t-test), data taken from [39].
B: No differences in HR capacity between transient and stable transfection in TNBC. For transient transfection a linearized pDR-GFP plasmid was used, in cells with stably integrated pDR-GFP an I-SceI coding plasmid was transfected. Transfection efficiency was measured with pEGFP-N1 in a parallel approach. After 24 hours, cells were harvested, and the percentage of GFP-positive cells determined by flow cytometry. HR capacity was calculated according to GFP-positive cells (pDR-GFP) and transfection efficiency (pEGFPN1), data were partially taken from [39].
C:Exemplary depictions of the results from FACS analysis of the HR-Assay of the MCF7 its respective RR clone. Exponentially growing cells were transfected with linearized DR-GFP plasmid (1 µg). Transfection effiency was determined by pEGFP-N1 (1 µg) in a parallel approach. Circular DRGFP (1µg) served as control. After 24 hours, cells were harvested, and the percentage of GFP-positive cells determined by flow cytometry, data were partially taken from [39].

## Slide 3
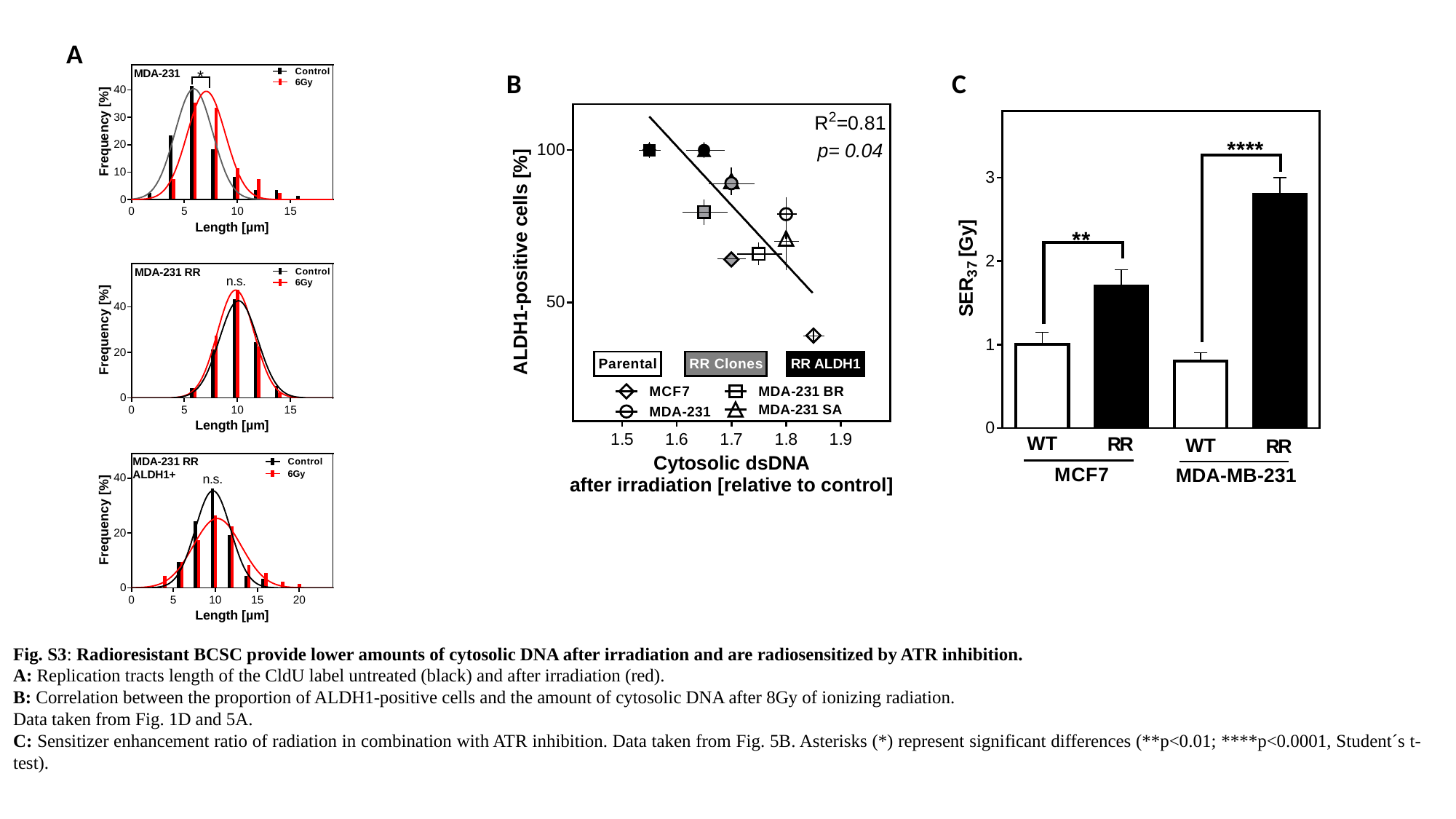

A
B
C
Fig. S3: Radioresistant BCSC provide lower amounts of cytosolic DNA after irradiation and are radiosensitized by ATR inhibition.
A: Replication tracts length of the CldU label untreated (black) and after irradiation (red).
B: Correlation between the proportion of ALDH1-positive cells and the amount of cytosolic DNA after 8Gy of ionizing radiation.
Data taken from Fig. 1D and 5A.
C: Sensitizer enhancement ratio of radiation in combination with ATR inhibition. Data taken from Fig. 5B. Asterisks (*) represent significant differences (**p<0.01; ****p<0.0001, Student´s t-test).

## Slide 4
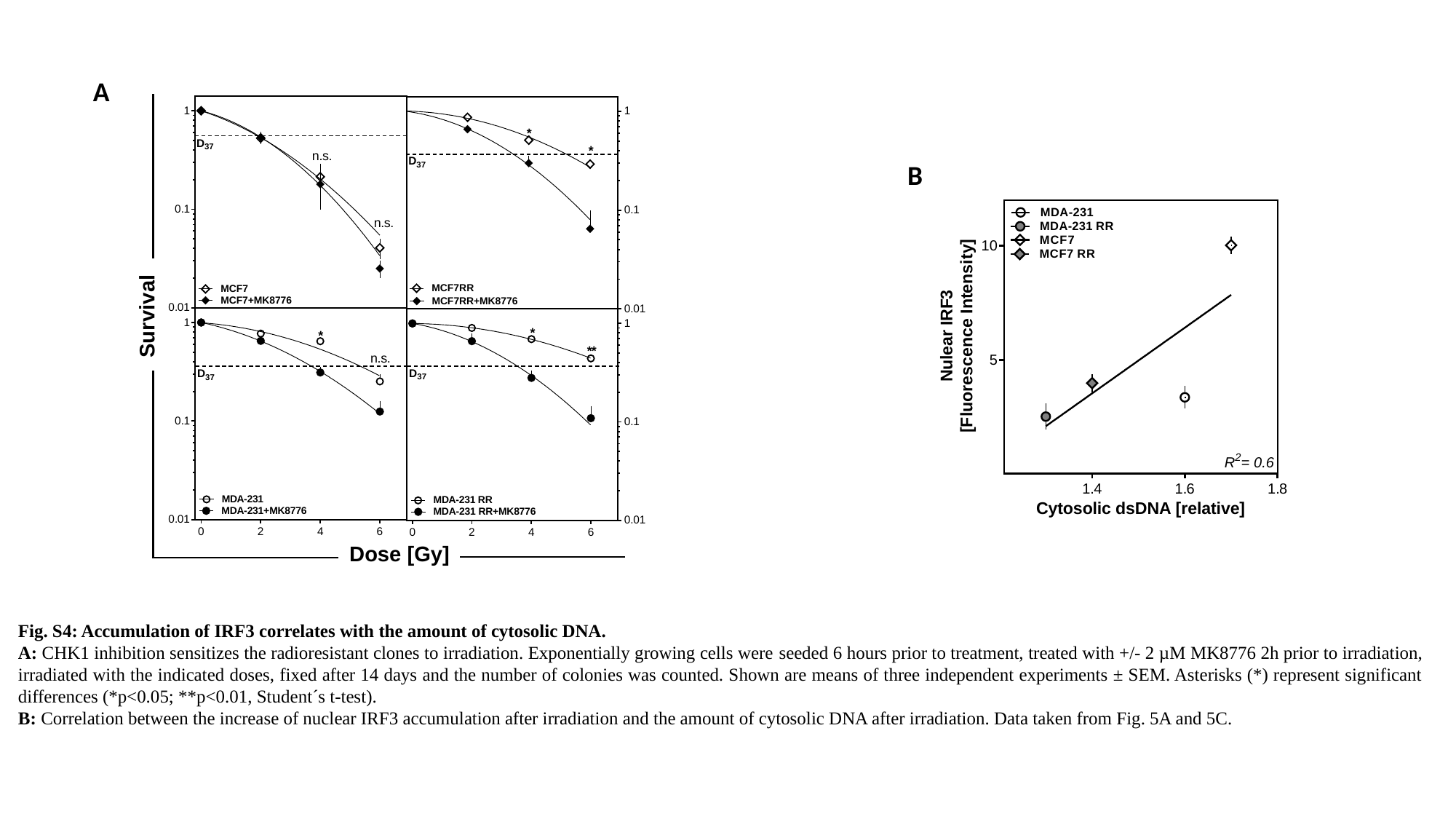

A
Survival
Dose [Gy]
B
Fig. S4: Accumulation of IRF3 correlates with the amount of cytosolic DNA.
A: CHK1 inhibition sensitizes the radioresistant clones to irradiation. Exponentially growing cells were seeded 6 hours prior to treatment, treated with +/- 2 µM MK8776 2h prior to irradiation, irradiated with the indicated doses, fixed after 14 days and the number of colonies was counted. Shown are means of three independent experiments ± SEM. Asterisks (*) represent significant differences (*p<0.05; **p<0.01, Student´s t-test).
B: Correlation between the increase of nuclear IRF3 accumulation after irradiation and the amount of cytosolic DNA after irradiation. Data taken from Fig. 5A and 5C.
